# Cigarette Smoking Among Adolescents With Alcohol and Other Drug Use Problems

**Published:** 2006

**Authors:** Mark G. Myers, John F. Kelly

**Affiliations:** Mark G. Myers, Ph.D., is a professor of psychiatry at the University of California, San Diego, California, and staff psychologist at the Veterans Affairs San Diego Healthcare System, San Diego, California. John F. Kelly, Ph.D., is associate director of the Addiction Research Program at Massachusetts General Hospital, Boston, Massachusetts, and assistant professor of Psychiatry at Harvard University, Boston, Massachusetts

**Keywords:** Alcohol and tobacco, alcohol, tobacco, and other drug (ATOD) use, abuse, and dependence, smoking, gateway drug, nicotine dependence, adolescent, youth, high-risk youth, alcohol and other drug use (AODU) treatment method, smoking cessation treatment, co-treatment, intervention, brief intervention, motivational interviewing, peer relations, treatment outcome

## Abstract

Cigarette and alcohol use often develop concurrently, and smoking is especially common among youth treated for alcohol and other drug (AOD) use disorders. Special considerations for adolescent smoking cessation treatment include peer influences, motivation, and nicotine dependence. Little research has addressed smoking cessation treatment for youth with AOD use disorders, but the few available studies suggest that tobacco cessation efforts are feasible and potentially effective for this population. Findings to date suggest that adolescents with AOD use disorders may benefit more from relatively intensive multicomponent programs rather than brief treatment for smoking cessation. Additional research is needed to further address the inclusion of tobacco-specific interventions for adolescents in AOD use disorder treatment programs.

Studies examining the origins of alcohol and other drug (AOD) use problems (i.e., AOD abuse and dependence) consistently find that cigarette smoking is closely related with AOD use. Because the use of psychoactive substances significantly escalates during the high school years (Johnston et al. 2006), most research in this area has focused on high school–aged youth (e.g., 14–18 years old). Although researchers have identified a broad range of factors that influence the initiation and progression of tobacco and AOD use, peer influences are particularly salient given the key developmental tasks of this period (e.g., establishing an identity separate from ones’ family). Ethnic and regional variations exist in the development of psychoactive substance use, yet adolescents typically start using cigarettes or alcohol prior to other drugs (Ellickson et al. 1992). Tobacco and alcohol, often referred to as “gateway drugs,” are among the first substances consumed by adolescents. This is likely influenced by their ready availability along with other sociocultural (e.g., peer influences, acculturation) and biological factors (e.g., family history of substance use disorders) (Vega and Gil 2005).

As with alcohol, adolescent cigarette smoking is strongly associated with illicit drug use (e.g., Eckhardt et al. 1994). In addition to more frequent use of illicit drugs, youth who consistently smoke throughout adolescence are at significantly greater risk for marijuana and other drug abuse or dependence (Vega and Gil 2005). Much of the research in this area has focused on concurrent use of cigarettes and alcohol, which predicts a variety of problems, both during adolescence and beyond. For example, it has been found that youth who smoke and drink have an increased risk of having difficulties at school, delinquency, and use of other drugs (Hoffman et al. 2001).

Adolescents who report consistent smoking and drinking have higher rates of deviant behavior and violence and are more likely to have legal and substance use problems in their 20s than those who consistently drink but do not regularly smoke (Orlando et al. 2005). The authors of the latter study noted, “…while it is common during adolescence to drink but not smoke, it is very unusual to smoke and not drink” (Orlando et al. 2005), suggesting that smoking is a reliable marker of adolescent alcohol use.

This article examines the prevalence of cigarette smoking among adolescents with AOD use problems, smoking cessation efforts in this population, and special considerations for adolescent smoking cessation treatment, including peer influences, motivation, and nicotine dependence. This article concludes with a brief review of studies evaluating smoking cessation treatment for adolescents with AOD use problems and a discussion of the implications of these findings.

## Prevalence of Cigarette Smoking Among Adolescents With AOD Use Problems

Research consistently demonstrates a link between adolescent smoking and psychiatric problems, in particular major depressive disorder, disruptive behavior disorders, and AOD use problems (e.g., Brown et al. 1996). Few studies have focused specifically on smoking in clinical samples^1^ of adolescents treated for AOD use problems. In an initial investigation of smoking among such adolescents (Myers and Brown 1994), 85 percent reported current (i.e., within the past 30 days) cigarette smoking at treatment entry, of whom 75 percent were daily smokers and 61 percent smoked 10 or more cigarettes daily. The frequency and intensity of smoking in this clinical sample was substantially higher than for adolescents in the general population. When these subjects were interviewed again 2 years following treatment, both prevalence of current smoking and average daily cigarette consumption by smokers had decreased, yet both remained very high. Heavier smokers at the time of treatment were more likely to report respiratory problems (e.g., bronchitis, pneumonia, and respiratory tract infections) at the 24-month followup, suggesting that health consequences from smoking may appear relatively early for these youth. In addition, rates of smoking were not associated with relapse to AOD use, suggesting that even adolescents abstinent from AODs remained at risk for continued cigarette use and related health problems.

In a subsequent report (Myers and Brown 1997), we examined the persistence of cigarette smoking 4 years following treatment for participants from the initial study, at which time these youth were, on average, 20 years old. Consistent with other reports indicating that cigarette use is a highly persistent behavior, 80 percent of those who smoked at the time of treatment were still smoking 4 years later. Those who had stopped smoking by the 4-year follow-up assessment reported lower rates of AOD involvement than those who continued to smoke. Thus, our studies to date underscore the fact that youth treated for AOD abuse are a heavy cigarette-smoking population, that smoking persists following treatment, and that smoking-related health problems already are evident during adolescence. Further, these initial studies provided suggestive evidence that smoking cessation does not detrimentally influence substance use outcomes. These findings highlight the importance of interventions to address tobacco use among these youth.

## Smoking Cessation Efforts

Information regarding self-initiated efforts to change smoking behaviors suggests that adolescent smokers frequently consider quitting smoking and, as such, may be amenable to intervention. Studies of general population and school samples consistently find that adolescents often want to stop smoking and frequently attempt smoking cessation yet seldom succeed in maintaining long-term abstinence. We recently examined smoking cessation among youth in treatment for substance abuse (Myers and Macpherson 2004). The majority (63 percent) of adolescents in the study had previously attempted to quit smoking yet reported difficulties staying abstinent––70 percent had returned to smoking within a month of quitting. The frequency and duration of smoking cessation efforts for this substance-abusing sample was comparable to findings with adolescent smokers from community-based studies (e.g., Burt and Peterson 1998; Pierce et al. 1998). Overall, adolescents tend to return to smoking relatively soon after cessation attempts, indicating the potential value of tobacco-focused interventions to enhance and support abstinence from smoking.

## Adolescent Smoking Cessation Treatment: Developmental Considerations

Developmental differences between adolescent and adult smokers pose significant challenges for designing effective youth-specific interventions (Mermelstein 2003). Interventions targeted at adolescents must thus include content appropriate to key developmental differences between youth and adults (*c.f.,* Milton et al. 2003). Peer influences and motivation for change are especially important considerations for any adolescent-focused tobacco use intervention and are particularly salient for youth with AOD problems. Adolescents also appear to differ from adults in characteristics of nicotine dependence (Colby et al. 2000). Another important issue to include in treatment is that the extent of dependence is found to influence cessation outcomes.

### Peer Influences

Studies consistently find that adolescents who associate with smoking peers have less success with quitting. The high smoking rates among AOD-abusing youth indicate that addressing peer influences may be particularly important with this population (Myers 1999). Adolescents strongly identify with their friends and peers, a phenomenon central to an adolescent’s development of a self-image distinct from one’s family (Greenberg et al. 1983). As such, the role of peers in adolescent smoking can be understood as part of an adolescent’s social identity (e.g., how youth view smoking in relation to self and others) and peer selection rather than solely as “peer pressure” (Ennett and Bauman 1994). For instance, cigarettes take on a social role by providing adolescent smokers a method for meeting and interacting with others. The social function of smoking may be particularly powerful for AOD-abusing adolescents, whether they currently are using substances or are abstaining, because their peer group likely will include many smokers. For example, being around smokers may lead to perceived pressure to smoke or may give rise to cravings.^2^ Because smoking is a normative behavior for AOD-involved youth, particular attention should be focused on youth perceptions regarding the acceptability of smoking, adolescents’ beliefs about the social role of cigarettes, beliefs regarding the relationship between smoking and other drug use (e.g., enhanced effects), and skills to resist temptations to smoke.

## Motivation

Motivation can be understood as a shifting state of desire to change, and, as such, promoting and enhancing the motivation to quit smoking is a central issue for both adolescent and adult treatment. Adolescents may have lower interest in quitting smoking than adults because they are less likely to have incurred subjectively noticeable negative consequences from smoking, and tobacco-related health problems tend to accrue gradually over a long period of time. For youth with AOD-related problems, the normative status of smoking may serve to dampen interest in quitting. In addition, those youth receiving treatment for a substance use disorder (and their parents) are likely to view smoking as a “lesser evil” compared with AOD use and, thus, a lower-priority issue. Incorporating client-centered motivational enhancement techniques^3^ as part of treatment is therefore likely to be particularly important when addressing smoking in the context of AOD use disorder treatment, because these youth may have little initial desire to change their smoking behavior (Myers 1999). As such, smoking intervention for AOD-abusing adolescents should address motivational obstacles to initial participation and include techniques to increase and sustain motivation for change.

## Nicotine Dependence

Most adolescents smoke cigarettes less often and in smaller quantities than adults. Despite lower levels of consumption, studies to date suggest that adolescent smokers, especially daily smokers, experience nicotine dependence, and the majority report experiencing withdrawal symptoms^4^ upon cessation (Colby et al. 2000). Available evidence also indicates that, as with adults, youth with higher levels of nicotine dependence have greater difficulty quitting smoking (Colby et al. 2000). Yet, the extent to which features of nicotine dependence are similar between adult and adolescent smokers is not yet well established. Treatment of adult smokers typically incorporates medication, such as nicotine replacement therapy (NRT) (e.g., transdermal nicotine patch). However, research has not yet established whether NRT and other medications are appropriate and effective for tobacco cessation among youth. Because a majority of AODabusing youth appears to be daily smokers, nicotine dependence should be addressed in the course of intervention. This can be accomplished by educating adolescents regarding the features of nicotine dependence (e.g., anticipating withdrawal symptoms), teaching strategies for coping with urges and withdrawal, and providing medication if appropriate (see Brown et al. 2003).

## Smoking Cessation Treatment for Adolescents With AOD Use Problems

Adolescent smoking cessation treatment has received growing attention over the past decade, yet few studies have focused on youth with AOD problems. We identified four published treatment studies relevant to this population, each of which addressed key developmental issues, including motivation, peer factors, and nicotine dependence.

### Project Ex

The largest study to date of smoking cessation with high-risk youth is Project Ex, an intervention designed for and delivered in continuation high schools^5^ (Sussman et al. 2001). Continuation schools serve high-risk youth, thus their students have a substantially higher prevalence of tobacco and AOD use than students in traditional high schools.

The intervention included eight sessions delivered over a 6-week period. A motivational approach was employed, such that students initially were not asked or required to quit but rather prepared for their quit attempt. Program content included examining reasons for smoking and quitting, education about the effects of tobacco and nicotine, coping skills, strategies for maintaining abstinence, and relapse prevention. It also included alternative medicine strategies and “talk shows” to make the intervention more appealing to youth.

The program was assessed by randomly assigning schools to one of three conditions: intervention alone, intervention with a school-as-community component^6^ (Sussman et al. 2001), and a no-treatment control. Three hundred and thirty-five smokers from 18 continuation high schools participated in the study. Follow-up assessment 5 months after the program quit day indicated a significant difference between treatment conditions; 17 percent of smokers in the treatment conditions reported having quit smoking for the last 30 days, compared with just 8 percent of those in the no-treatment control condition. Project Ex is one of the first studies to demonstrate the benefits of smoking cessation interventions with high-risk youth. Although this intervention’s content was developed and implemented with high-risk youth rather than youth diagnosed with AOD use disorders, its content may well apply to AOD-abusing youth.

### Motivational Intervention for Adolescents With Psychiatric and Substance Use Disorders

A recently completed treatment study compared the effectiveness of an intervention designed to enhance motivation to quit with a brief advice condition for smoking cessation in adolescents admitted for psychiatric hospitalization for any axis I condition other than a psychotic disorder (e.g., schizophrenia) (Brown et al. 2003). The motivational intervention consisted of two 45-minute sessions. The first session involved examination of the pros and cons of smoking and quitting, followed by feedback regarding peer influences, nicotine dependence, adolescent attitudes toward smoking, and costs of smoking. The second session incorporated feedback about the effects of smoking on appearance, pulmonary health, and measurement of lung function. This session ended with discussion of a plan for changing cigarette use. Participants in the motivational interview condition also received a manual that outlined skills for preventing relapse to smoking and that described strategies for coping with negative moods. In addition, participants received up to six brief counseling phone calls during the initial 6 months following hospitalization and parents received up to four phone counseling sessions. Finally, in order to further address nicotine dependence, eligible participants in this motivational intervention condition received up to two 8-week supplies of transdermal nicotine patch therapy.

The brief advice condition was designed in accordance with current clinical guidelines for smoking cessation, and participants were given 5 to 10 minutes of advice about quitting smoking. Participants received a clear recommendation to quit smoking, information about the negative health effects of smoking, and advice for quitting (e.g., setting a quit date). Eligible participants also received a single course of transdermal nicotine patch therapy.

The study included 191 adolescents who were randomly assigned to receive either motivational enhancement or brief advice and were followed for 12 months after discharge from the hospital. Several outcomes were compared across treatment conditions, including proportion attempting to quit smoking, duration of longest quit attempt, smoking abstinence rates, and smoking rates (cigarettes per day). Overall, no significant differences were observed between the treatment conditions on frequency of quit attempts or rates of abstinence, suggesting that the motivational intervention had no more effect on smoking than brief advice. Cessation rates reported for this study (9 to 14 percent of participants in each condition were abstinent for the prior week at follow-up assessments) were comparable to those of the no-treatment conditions of the other studies reviewed herein, suggesting that this brief motivational intervention had little effect on smoking in this clinical population. Although a similar intervention with a nonclinical sample of adolescents has shown positive findings (Colby et al. 2005), adolescents with concurrent substance use and other psychiatric disorders may require more intensive intervention than provided in this study.

### Cigarette Smoking Intervention for Adolescents in AOD Abuse Treatment

In response to the need for smoking interventions targeted to youth with AOD use problems, we conducted a series of studies on this issue. The first study focused on designing and implementing a smoking intervention with this population (Myers et al. 2000). Subsequently, we conducted a controlled comparison study of the previously developed intervention (Myers and Brown 2005).

The initial study (Myers et al. 2000) aimed to produce a treatment manual and demonstrate the feasibility of providing a tobacco-focused intervention in the context of treatment for AOD use disorders. Design of the intervention was based on available research regarding influences on adolescent smoking cessation and persistence, developmental issues, and factors specific to AOD-abusing youth. Each version of the intervention addressed these primary considerations, and subsequent applications were modified in response to prior experience and participant feedback. In keeping with the client-centered, motivational approach underlying the intervention, participants were asked to set their own goals for change, with smoking cessation representing one of several possible outcomes. The final version of the intervention consisted of six, weekly, hour-long group sessions integrated within existing programs for treatment of adolescent AOD use disorders. Content included strategies for increasing motivation for change, training in behavioral strategies for reducing cigarette use, skills for managing urges to smoke, and preventing relapse to smoking. Social aspects of adolescent smoking were addressed, with an emphasis on obtaining peer support for quitting smoking and skills for refusing offers of cigarettes. Commonalities and differences between adolescent experiences with AOD use versus cigarette use were also discussed.

Thirty-five adolescents participated in this pilot project and were followed for 3 months following completion of the smoking intervention. Analyses found that 3 months after treatment, 6 of 28 (21 percent; 17 percent of original sample) participants reported abstinence from smoking for at least the prior 7 days. Forty-six percent of participants reported at least one quit attempt following participation. Further, no relationship was found between attempting to quit smoking and extent of AOD use. These findings provided initial support for the feasibility of this approach, and evidence that providing a client-centered, motivation-enhancing, tobacco intervention in the context of AOD treatment was not detrimental to AOD-related treatment outcomes for adolescents.

**Figure 2:**
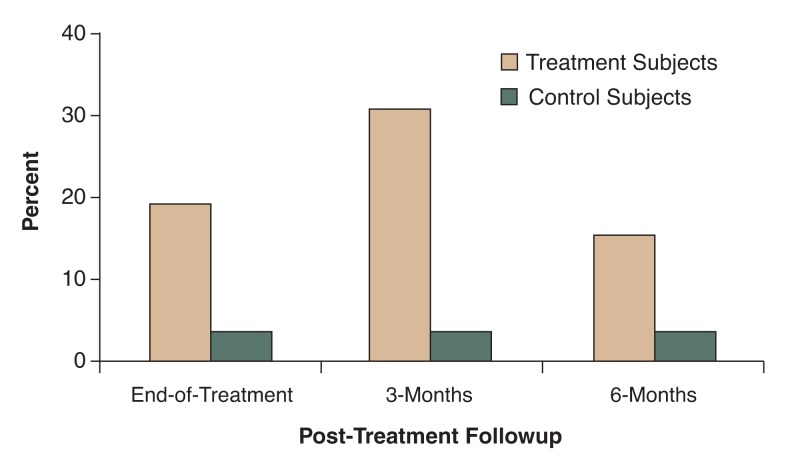
Proportion of adolescents abstinent from smoking for at least 7 days across treatment and control conditions at the end of treatment and at 3- and 6-months’ followup. Compared with individuals in the control condition, treatment participants were more likely to report past-week abstinence from smoking at each of the three followups. SOURCE: Myers and Brown 2005.

The efficacy of this intervention was subsequently assessed in a controlled outcome study (Myers and Brown 2005). Fifty-four adolescents recruited from AOD use disorder treatment programs were assigned either to receive the intervention or to a waitlist control condition. Those assigned to the control condition were offered access to the intervention after completing study participation. Participants were followed for 6 months after completing the smoking intervention and were compared across conditions on rates of quit attempts and abstinence from smoking. Analyses were conducted employing an intent-to-treat approach, whereby all participants who entered the study were included, with those lost to follow-up considered as not having attempted cessation and not abstinent. No differences were found across groups for frequency of cessation attempts. However, compared with individuals in the control condition, treatment participants were more likely to report pastweek abstinence from smoking at the end of treatment (4 percent versus 38 percent, respectively), at the 3-month follow-up (4 percent versus 31 percent, respectively), and at the 6-month follow-up (4 percent versus 15 percent respectively) (see Figure 2). That a statistically significant difference was found only for abstinence at 3 months posttreatment may reflect the small sample size and consequent limited statistical power. This study provided initial evidence for the efficacy of a smoking cessation intervention delivered in the context of adolescent substance use disorder treatment.

### Summary of Findings

The evolution of smoking treatment for adolescents with AOD-related problems is in its early stages. The few relevant treatment studies indicate that tobacco use interventions for this population are feasible and potentially effective. Findings to date suggest that adolescents with AOD use disorders may benefit more from relatively intensive multicomponent programs rather than brief treatment for smoking cessation. It is noteworthy that all of the treatments reviewed focused on clientcentered approaches to motivating changes in smoking behavior and, in contrast with standard adult treatment, did not require cessation. Motivational approaches thus could be especially important for engaging adolescents in treatment. The studies showing significant effects included interventions delivered in group rather than individual sessions. This finding may reflect that adolescent social factors may be more salient and effectively addressed in a group, rather than individual, format. Finally, offering a tobacco-focused intervention as an integral part of AOD treatment may serve to reduce barriers to participation and alter normative attitudes regarding tobacco use.

These conclusions must be tempered by considering the limitations of the studies reviewed. Project Ex, the study with the strongest outcomes, did not specifically focus on adolescents diagnosed with substance use disorders. As such, further research is needed to establish whether this same program would prove effective among youth with AOD use disorders. Generalizing the findings of these studies is further complicated by gender and ethnic composition differences across studies. Finally, the two studies conducted with youth treated for AOD use disorders included small samples and as such require replication with larger samples.

## Implications for Adolescent AOD Use Disorder Treatment

Cigarette and alcohol use share common etiological factors and often develop concurrently. Smoking is especially common among youth treated for AOD use disorders and appears to persist at least into early adulthood. Despite the potentially severe health consequences of concurrent tobacco and alcohol use and the possible benefits of early intervention, little research has addressed treatments targeted at this population. The few available studies suggest the feasibility and utility of addressing tobacco use among youth with AOD problems. When considered in concert with findings that client-centered adolescent smoking cessation efforts do not appear to detract from AOD use outcomes, the current evidence is cause for optimism. However, the dearth of studies and limitations of current research serve to emphasize the need for additional research to address the inclusion of tobacco-specific interventions for adolescents in AOD use disorder treatment programs.

## Figures and Tables

**Figure 1:**
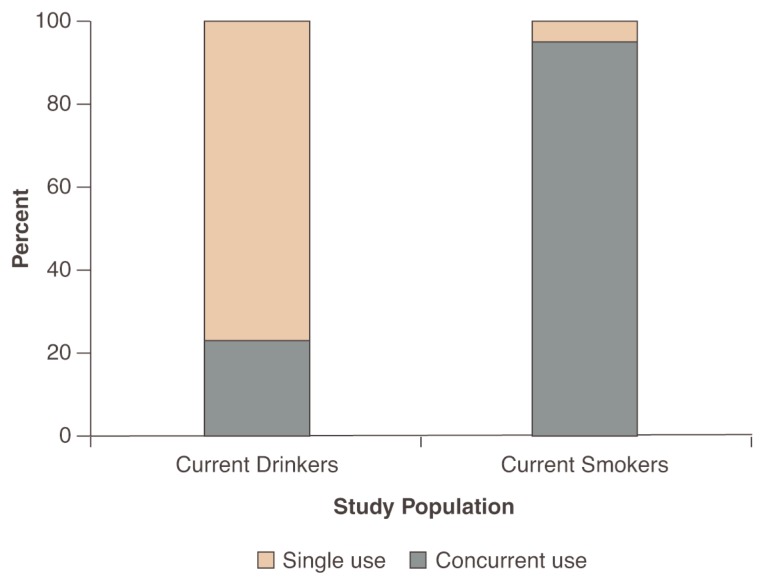
The overlap between adolescent alcohol and cigarette use is illustrated by data from Statewide surveys of 7th to 12th grade students in New York State. Evaluation of concurrent use of alcohol and cigarettes indicated that approximately one-third of current drinkers smoked, whereas approximately 95 percent of current smokers used alcohol. SOURCE: Hoffman et al. 2001.

## Footnotes

1 A clinical sample refers to study participants recruited from inpatient or outpatient treatment settings.

2 A strong perceived desire or urge to smoke.

3 Motivational enhancement techniques refer to therapeutic strategies designed to increase the likelihood of an individual engaging in and maintaining behavior change. Motivational interventions incorporate the client-centered principles of empathy, acceptance, and positive regard.

4 Common withdrawal symptoms that occur within 24 hours after stopping smoking include irritability, anxiety, poor concentration, insomnia, and depression.

5 Continuation high schools are part of the California public high school system and serve youth who are diverted from the traditional high schools for reasons such as behavior problems and poor academic performance.

6 The community component was designed to promote school-wide changes in attitudes toward smoking as a means for facilitating cessation efforts.
